# Thromboelastography (platelet contribution to clot strength) for the assessment of platelet residual function

**DOI:** 10.1186/cc11039

**Published:** 2012-03-20

**Authors:** D Haxhiademi, S Parri, A Cerillo, P Del Sarto, R Paniccia, D Prisco

**Affiliations:** 1 Fondazione Toscana G. Monasterio, Massa, Italy; 2 Thrombosis Centre, University of Florence, Italy

## Introduction

In the early postoperative period after cardiac surgery, platelet dysfunction is one of the main causes of excessive bleeding; there is still controversy regarding the timing of antiplatelet therapy discontinuation [1]. The Clinical Practice Guidelines of the Society of Thoracic Surgeons recommend that point-of-care (POC) testing may help identify patients who can safely undergo urgent operations [2]. This study was designed to test the relationship between platelet function as revealed by POC tests and postoperative bleeding in patients that undergo cardiac surgery without suspending thienopyridines at least 5 days prior to surgery.

## Methods

Adult patients scheduled for cardiac operations in which thienopyridines were not discontinued at least 5 days before surgery were included. From November 2010 to February 2011, 20 patients were enrolled in this pilot study. Samples were taken before induction of anesthesia (T0) and 2 hours after arrival in the ICU (T1). Standard laboratory tests and the following POCs were performed: multiple electrode aggregometry (MEA), PFA 100 and thromboelastography (TEG). Functional fibrinogen level (FFL) is a recent modification of TEG used to investigate the function of fibrinogen [3]. We used the combination of TEG and FFL to detect platelet contribution to the clot strength.

## Results

There was no significant association between bleeding at 4, 6 and 12 hours and any of the preoperative tests. There was no significant association between bleeding at 4, 6 and 12 hours and any of the standard laboratory tests. Platelet contribution to clot strength (%pltMA) detected by TEG showed a significant association with postoperative blood loss (at 4, 6 and 12 hours, respectively *P *= 0.02, *P *= 0.02, *P *= 0.01).

## Conclusion

Our data confirm the utility of perioperative evaluation of platelet contribution to clot strength evaluated by TEG. It helps to understand the mechanism behind the surgical bleeding and reduce empirical transfusions.
